# Identification of Critical Amino Acid Residues of a Two-Component Sensor Protein for Signal Sensing in *Porphyromonas gingivalis* Fimbriation via Random Mutant Library Construction

**DOI:** 10.3390/pathogens13040309

**Published:** 2024-04-10

**Authors:** Haruka Iida, Kiyoshi Nishikawa, Takuma Sato, Misuzu Kawaguchi, Ken Miyazawa, Yoshiaki Hasegawa

**Affiliations:** 1 Department of Orthodontics, School of Dentistry, Aichi Gakuin University, 2-11 Suemori-dori, Chikusa-ku, Nagoya 464-8651, Japan; 2 Department of Microbiology, School of Dentistry, Aichi Gakuin University, 1-100 Kusumoto-cho, Chikusa-ku, Nagoya 464-8650, Japan; yhase@dpc.agu.ac.jp

**Keywords:** two-component system, FimS sensor protein, signal sensing, FimA fimbriae, random mutagenesis, error-prone PCR, inverse PCR, conjugal transfer, amino acid substitution, functional mapping

## Abstract

*Porphyromonas gingivalis* (*Pg*) utilizes FimA fimbriae to colonize the gingival sulcus and evade the host immune system. The biogenesis of all FimA-related components is positively regulated by the FimS–FimR two-component system, making the FimS sensory protein an attractive target for preventing *Pg* infection. However, the specific environmental signal received by FimS remains unknown. We constructed random *Pg* mutant libraries to identify critical amino acid residues for signal sensing by FimS. Optimized error-prone polymerase chain reaction (PCR) was used to introduce a limited number of random mutations in the periplasmic-domain-coding sequence of *fimS*, and expression vectors carrying various mutants were generated by inverse PCR. More than 500 transformants were obtained from the *fimS*-knockout *Pg* strain using the *Escherichia coli*–*Pg* conjugal transfer system, whereas only ~100 transformants were obtained using electroporation. Four and six transformant strains showed increased and decreased *fimA* expression, respectively. Six strains had single amino acid substitutions in the periplasmic domain, indicating critical residues for signal sensing by FimS. This newly developed strategy should be generally applicable and contribute to molecular genetics studies of *Pg*, including the elucidation of structure–function relationships of proteins of interest.

## 1. Introduction

**Figure 1:**
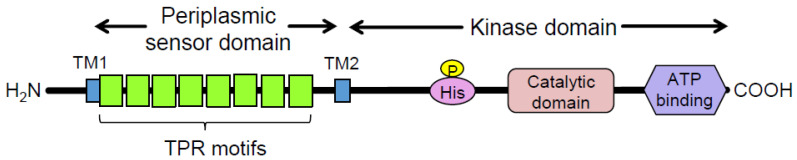
Domain organization of the FimS sensor histidine kinase. TPR, tetratricopeptide repeat. Eight TPR motifs are predicted by TPRpred [10] to be clustered in the periplasmic sensor domain of FimS. TM1/2, transmembrane helices.

*Porphyromonas gingivalis* (*Pg*) is a Gram-negative oral anaerobe strongly associated with adult periodontitis [1]. Among the known virulence factors of *Pg*, gingipains, trypsin-like proteinases, play a crucial role in bacterial housekeeping and infection and exhibit multiple pathogenic activities [2]. They have garnered substantial attention as potential targets for therapeutic agents against periodontitis and associated systemic diseases [3]. Another major virulence factor of *Pg* is FimA fimbriae, which are important for the colonization of the gingival sulcus and evasion of the host immune system [4]. Fimbriae biogenesis is positively regulated at the transcriptional level by the FimS–FimR two-component system (TCS) [5]. Specifically, the putative inner-membrane-localized sensory protein *FimS* comprises two functional domains: a well-conserved histidine kinase domain at the C-terminus and a periplasmic sensor domain between the two transmembrane regions at the N-terminus (Figure 1) [6]. FimS has been proposed to function as a homodimer, similar to many known histidine kinases, when it receives a unique environmental signal [7]. The autokinase activity of the intracellular domains of the FimS dimer activates the cognate transcriptional regulator FimR, which in turn upregulates transcription of the *fim* gene cluster and produces all components of the fimbriae, including the major subunit protein, FimA. Disruption of either *fimS* or *fimR* loci results in a clear afimbriation phenotype without killing the mutants [6,8]. The inhibition of signal sensing by FimS could suppress the production of all components of FimA fimbriae, which could drastically reduce the periodontal pathogenicity of *Pg* as a keystone pathogen among the disease-provoking periodontal microbiota [9]. Therefore, FimS could be a promising target for the precise suppression of major virulence factors in *Pg*, delaying the emergence of drug-resistant strains. However, the specific environmental signals to which the TCS responds remain unknown. The periplasmic sensor domain of FimS contains multiple tetratricopeptide repeat (TPR) motifs (Figure 1) [10], which are known to provide interfaces for protein–protein interactions and bind to various types of compounds, including peptides and polyunsaturated fatty acids [11]. Therefore, identifying the amino acid residues within this sensor that are essential for signal sensing would provide useful information to determine the natural environmental signals for FimS. In turn, the identification of these signals could inform the design of a novel antagonist that effectively inhibits the biogenesis of FimA fimbriae.

The FimS periplasmic domain is composed of 338 amino acids (GenBank No. BAF63427). Therefore, an efficient strategy is required for the comprehensive identification of these important amino acid residues. In this study, we utilized a gene complementation system to construct random mutant libraries of *Pg*, which were applied for the identification of amino acid residues within the periplasmic domain of FimS that are critical for its sensory function. The specific mutation sites in the periplasmic-domain-coding region were identified and linked to phenotypes in terms of their impact on FimA fimbriae expression.

## 2. Materials and Methods

### 2.1. Bacterial Strains and Culture Conditions

The bacterial strains used in this study are listed in Table 1. The *Pg*-type strain ATCC 33277 was used as the FimA-fimbriae wild-type strain. AGFS1 is a *fimS*-knockout (KO) mutant strain lacking FimA fimbriae. All *Pg* strains were grown on trypticase soy agar plates supplemented with defibrinated rabbit blood, hemin, and menadione (BAPHK) or cultured in supplemented trypticase soy broth medium (sTSBHK) [5], with appropriate antibiotics, if necessary, under standard anaerobic growth conditions (10% CO_2_, 5% H_2_, 85% N_2_) established using either the AnaeroPack-Anaero (Mitsubishi Gas Chemical, Tokyo, Japan) or Anoxomat III (Advanced Instruments, Norwood, MA, USA) system. *Escherichia coli* strains DH5α and BL21-Gold were used as hosts for general cloning procedures, including plasmid construction, and strain S17-1 was used as a host for the conjugal transfer of *E. coli*-*Pg* shuttle vector plasmids [12]. Cells were grown on Luria–Bertani (LB) agar plates or in LB broth supplemented with appropriate antibiotics, if necessary, under standard culture conditions.

### 2.2. Overview of the Experimental Design

**Figure 2:**
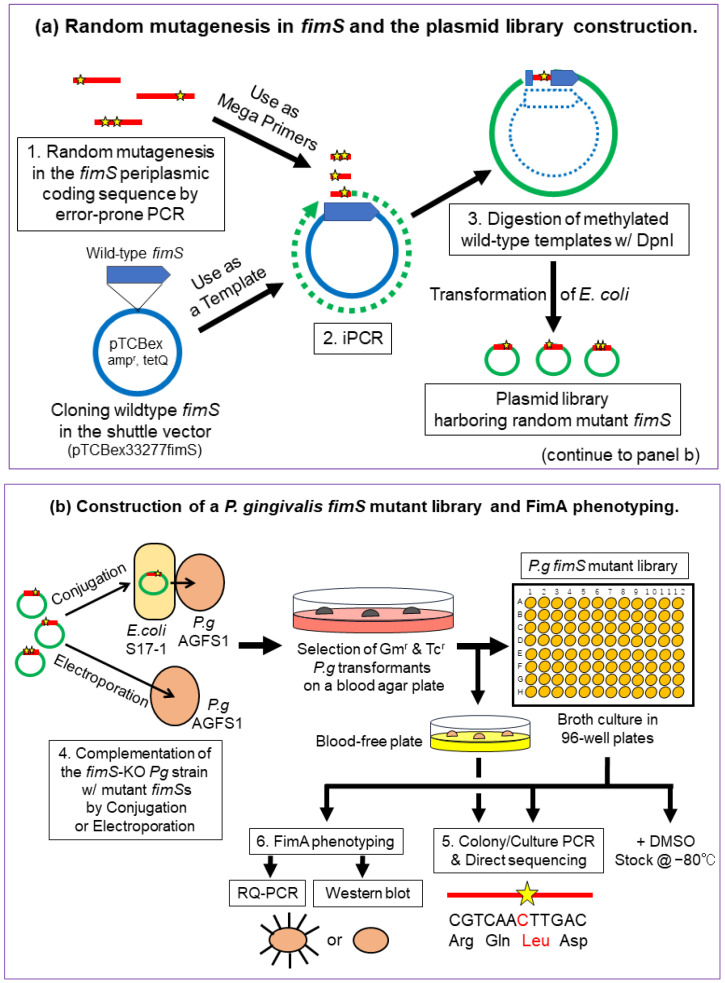
Strategy for random mutagenesis in the FimS periplasmic-domain-coding sequence. (**a**) Step 1, amplification of the 1.1 kb periplasmic coding sequence harboring random mutations (indicated as stars) by error-prone PCR. Step 2, exchange the wild-type sequence with the mutant using inverse PCR (iPCR). Step 3, digestion of methylated wild-type template plasmid with DpnI. (**b**) Step 4, complementation of the *fimS*-knockout (KO) *P. gingivalis* strain, AGFS1, with the mutant plasmid (iPCR products) by conjugation or electroporation. Step 5, screening of amino acid-substituted FimS clones by colony/culture PCR and direct sequencing. Step 6, FimA phenotype analyses (RQ-PCR and Western blotting).

The experimental strategy for creating and identifying amino acid substitutions within the FimS periplasmic domain is shown in Figure 2. The main steps included (1) amplification of the 1.1 kb periplasmic coding sequence with flanking regions of partial TM1/2 sequences harboring a random mutation introduced by error-prone polymerase chain reaction (PCR) [15,16], (2) exchanging the wild-type sequence with the mutant sequence by inverse PCR (iPCR) [17], (3) digestion of the methylated wild-type template plasmid with DpnI (Figure 2a), (4) complementation of the *fimS*-KO *Pg* strain (AGFS1) with the mutant plasmid (iPCR products) by conjugation or electroporation, and (5) sequencing and phenotype analyses of the clones at the transcriptional [real-time quantitative PCR (RQ-PCR)] and translational (Western blot) levels (Figure 2b).

### 2.3. Construction of Pg Random Mutant Libraries

#### 2.3.1. Error-Prone PCR

The 50 μL reaction mixture contained 0.2 mM of each dATP and dGTP, 1 mM of each dCTP and dTTP [18], 0.2 μM of the FimSperi5/3 primer set (Table 1), 118 ng of the wild-type full-length *fimS* gene as template DNA, 10 U Paq5000 DNA polymerase (Agilent, Tokyo, Japan), and 1× reaction buffer. The reaction conditions were as follows: initial denaturation at 95 °C for 2 min; followed by 32 cycles of denaturation at 95 °C for 20 s, annealing at 60 °C for 20 s, and extension at 72 °C for 40 s; and an additional extension step at 72 °C for 1 min. The 1.1 kb (1097 bp) amplicons were analyzed by electrophoresis on a 1% agarose gel and then extracted from the gel using DNA Clean & Concentrator-5 (Zymo Research, Irvine, CA, USA). A total of 1964 ng (49.1 ng/μL) of the purified fragments was obtained from the 40 μL reaction system, and the obtained purified fragments were used as megaprimers in the subsequent iPCR step (Supplementary Materials Figure S1).

#### 2.3.2. iPCR

The periplasmic-domain-coding region of wild-type *fimS* was exchanged with randomly mutated sequences by iPCR using a high-fidelity DNA polymerase. The 50 μL reaction mixture contained 100 ng of the wild-type template plasmid pTCBex33277*fimS* (Table 1, Supplementary Materials Figure S2) as a template, 500 ng of the purified error-prone PCR amplicons obtained as described above as megaprimers, 0.2 mM dNTPs, 2.5 mM MgSO_4_, 1× reaction buffer, and 2 units of KOD-plus DNA polymerase (TOYOBO, Osaka, Japan). The reaction conditions were as follows: initial denaturation at 94 °C for 2 min; followed by 25 cycles of denaturation at 95 °C for 50 s, annealing at 60 °C for 50 s, and extension at 68 °C for 21 min; and a final additional extension step at 68 °C for 10 min. The wild-type template plasmids (pTCBex33277*fimS*; methylated in the *E. coli* host) were digested using 40 units of DpnI (New England Biolabs, Ipswich, MA, USA) for 2 h at 37 °C.

#### 2.3.3. Evaluation of the Mutation Rate

Ten microliters of the DpnI-treated iPCR products was mixed with 200 μL of *E. coli* BL21-Gold chemical competent cells (Agilent, Tokyo, Japan) for heat-shock transformation. A total of 514 colonies were grown on two LB agar plates supplemented with 100 μg/mL ampicillin. Eight randomly selected colonies were subjected to colony PCR, followed by direct DNA sequencing to evaluate the mutation rate.

### 2.4. Introduction of the Mutant Plasmids into the fimS-KO Pg Strain by the E. coli–Pg Conjugal Transfer System or Electroporation

#### 2.4.1. Electro-Transformation of *E. coli* S17-1 with iPCR Products

Five microliters from the 50 μL iPCR system described above were combined with 100 μL of S17-1 cells suspended in 10% glycerol water, and then electroporation was conducted using a 2 mm gapped cuvette and an ECM 630 electroporator (BTX Harvard Apparatus, Hollistion, MA, USA). The initial setting parameters were as follows: Vmax, 2500 V; resistor, 200 Ω; capacitor, 25 μF. The actual delivered peak voltage and time constant were 2410 V and 4.2 ms, respectively. After the pulse, 1 mL of SOC broth was added, and the cell culture was shaken at 37 °C for 1 h. The culture was then streaked on two LB agar plates supplemented with 100 μg/mL ampicillin and grown overnight at 37 °C. A single electro-transformation obtained a total of 1140 clones from the S17-1 colonies. These colonies were grown until their diameter reached at least 1 mm and were then mixed and suspended in 4 mL of SOC broth per plate using a cell spreader. The cell suspension containing multiple clones was supplemented with glycerol to a final concentration of 15%, and 200 μL aliquots were stored at –80 °C for their subsequent use in conjugation as plasmid donor cells.

#### 2.4.2. Conjugal Transfer of the Mutant Plasmid Library from S17-1 to *fimS*-KO *Pg* (AGFS1)

The recipient strain of the mutant plasmid, *Pg* strain AGFS1, was anaerobically cultured in the sTSBHK (−) broth to an optical density at 600 nm (OD_600_) of 0.3, which was designated as the time point of 8 h before conjugation. The donor strain of the mutant plasmid, *E. coli* strain S17-1, which was transformed with iPCR amplicons as described above, was prepared as follows: A frozen 200 μL aliquot was thawed and precultured in 2 mL of LB (−) broth at 37 °C for 1 h with continuous shaking for an additional 45 min with supplementation of final concentrations of 100 μg/mL ampicillin and 50 μg/mL trimethoprim. At the time point of 1 h before conjugation, 200 μL of the S17-1 culture was diluted with 4 mL LB (−) (i.e., 20× dilution, OD_600_ = 0.12). The conjugation procedure was started when the OD_600_ of the recipient AGFS1 strain and the donor S17-1 strain reached 1.03 and 0.24, respectively. First, 2 mL of the S17-1 culture was centrifuged at 5000× *g* for 5 min, and the cell pellet was resuspended in 2 mL of the AGFS1 culture. The combined culture was centrifuged at 5000× *g* for 5 min, and then the mixed cell pellet was removed and spotted on an sBAPHK (−) plate and anaerobically incubated overnight at 37 °C. After 13.5 h of mating, the mixed cell pellet was removed and re-streaked onto two sBAPHK plates supplemented with final concentrations of 1 μg/mL tetracycline and 100 μg/mL gentamycin. These plates were incubated under standard anaerobic conditions for 5–7 days until the plasmid-containing *P. gingivalis* AGFS1 cells selectively grew as black-pigmented colonies.

#### 2.4.3. Introduction of the Mutant Plasmids into the *fimS*-KO *Pg* Strain by Electroporation

The pTCBexdm plasmid, which is a pTCBex derivative with its Mob region deleted for resizing and improving subcloning efficiency (Supplementary materials Figure S2) [14], harboring random mutant *fimS* was prepared at 2.4 μg by the transformation of *E. coli* DH5α with the iPCR products (see Section 2.3.2) and mixed with broth-cultured AGFS1 cells in 100 μL of 10% glycerol water. Electroporation was conducted as described above in Section 2.4.1. The actual delivered peak voltage and time constant were 2390 V and 4.7 ms, respectively. After the pulse, 1 mL of sTSBHK broth was added, and the cell suspension was moved to a 15 mL conical plastic tube. The cells were maintained at 37 °C under standard anaerobic conditions for 24 h and then streaked onto two sBAPHK plates supplemented with a final concentration of 1 μg/mL tetracycline. These plates were anaerobically incubated at 37 °C for more than 5 days.

#### 2.4.4. Subculture and Preparation of the Frozen Stocks of pTCBex-Introduced *Pg* Strains

The pTCBex-introduced *Pg* strains were maintained and subcultured on an sBAPHK blood agar plate supplemented with 1 μg/mL tetracycline by inoculation with sterilized toothpicks. For longer storage, a cell chunk of each clone was suspended in sTSBHK supplemented with 5–7% dimethyl sulfoxide and stored frozen at –80 °C.

### 2.5. Colony PCR and Direct Sequencing of the FimS Periplasmic-Domain-Coding Region

Twenty microliters of the colony PCR mixture contained 10 μL KAPA HiFi HotStart ReadyMix (2×) (Kapa Biosystems, Cape Town, South Africa) and 0.5 μM of the *FimS*peri5/3 primer set (Table 1). *Pg* transformant colonies on sBAPHK blood agar plates were recultured on a blood-free trypticase soy agar plate (sTSAHK) supplemented with 1 μg/mL tetracycline to reduce heme acquisition and improve amplification efficiency during PCR and then rubbed against the inner wall of the PCR tubes as templates using sterilized toothpicks. Alternatively, 1 μL sTSBHK culture of each clone was used as a template. The reaction conditions were as follows: initial denaturation at 95 °C for 1 min; followed by 27 cycles of denaturation at 95 °C for 30 s, annealing at 55 °C for 30 s, and extension at 72 °C for 1 min 30 s; and a final additional extension step at 72 °C for 1 min. The 1.1 kb PCR amplicons were analyzed by 1% agarose electrophoresis and purified using DNA Clean & Concentrator-5 (Zymo Research, Irvine, CA, USA) according to the manufacturer’s instructions. PCR amplicons were sequenced using the BigDye Terminator v3.1 Cycle Sequencing Kit (Applied Biosystems, Foster City, CA, USA) and an ABI Prism 3130-Avant Genetic Analyzer (Applied Biosystems, Japan). Only two primers were used for sequencing each clone, FIMSSFW3.5 and FIMSSFW4rv (Table 1), which were designed to extend in opposite directions from the center of the target sequence and completely covered the 1.1 kb of the whole periplasmic-domain-coding sequence to be analyzed for mutations.

### 2.6. Preparation of RNA and RQ-PCR

Broth-cultured *Pg* (OD_600_ = 1.5–2.0, mainly 1.7) was aliquoted into 900 μL samples and centrifuged at 12,000× *g* for 10 min at 4 °C. Total RNA was extracted using the PureLink RNA Mini kit (Invitrogen/Thermo Fisher Scientific, Carlsbad, CA, USA) according to the manufacturer’s instructions with minor modifications. The cell pellet was resuspended in a mixture containing 100 μL of Tris EDTA (TE) (pH 8.0), 0.5 μL of 10% sodium dodecyl sulfate (SDS), 350 μL of lysis buffer, and 100 μL of zirconia beads (Invitrogen) and then vortexed for 5 min at 4 °C. After centrifugation at 12,000× *g* for 5 min, the cell lysate was processed according to the manufacturer’s instructions. Fifty microliters of the extracted total RNA solution was treated with TURBO DNA-free reagent (Invitrogen/Thermo Fisher Scientific) according to the manufacturer’s instructions. Reverse transcription of the purified RNA and cDNA synthesis were performed using the SuperScript III First-Strand Synthesis System for RT-PCR (Invitrogen/Thermo Fisher Scientific) according to the manufacturer’s instructions.

RQ-PCR was performed using the 7900HT Fast Real-Time PCR System (Applied Biosystems). Forty microliters of a reaction mixture containing 500 ng of cDNA and either a *fimA*-specific or 16S rRNA primer set (see Table 1) was prepared using the GoTaq qPCR Master Mix (Promega, Madison, WI, USA) according to the manufacturer’s protocol. Reactions were performed in triplicate for each cDNA–primer combination as technical replicates. The thermal cycling conditions were as follows: initial denaturation at 95 °C for 2 min, followed by 40 cycles of denaturation at 95 °C for 15 s, annealing at 56 °C for 15 s, and extension at 72 °C for 1 min. The expression level of *fimA* was normalized to that of the endogenous 16S rRNA in each sample. Relative *fimA* expression was quantified using the comparative cycle threshold (C_T_) method [19]. The wild-type or G2 strain (an iPCR amplicon-transformed strain with no amino acid substitutions) served as the calibrator for expression analysis.

### 2.7. Western Blot Analysis

Rabbit anti-FimA anti-serum was raised against a synthetic peptide, 327FA301 (CITGPGTNNPENPITES; Sigma-Aldrich Japan, Ishikari, Japan), selected from the *Pg* ATCC 33277 FimA amino acid sequence. The anti-serum was used as the primary antibody in the Western blot analysis. Phenotypic analyses of the *fimS*-complemented strains harboring amino acid substitutions were performed by Western blot using whole-cell lysates from each clone. Broth-cultured *Pg* (OD_600_ = 1.5–2.0, mainly 1.7) was aliquoted to 900 μL samples and centrifuged at 12,000× *g* for 10 min at 4 °C. Cell pellets were resuspended in 100 μL of 1× SBuffer (33 mM Tris-HCl [pH 6.8], 1% SDS) and mixed with an equal volume of phosphate-buffered saline containing 1 mM Tosyl-L-lysyl-chloromethane hydrochloride (TLCK) and 1 mM leupeptin. The cell suspension was briefly sonicated and incubated at 100 °C for 15 min. After centrifugation, the protein concentration in the supernatant was determined by measuring the OD_280_ value using a NanoDrop ND-1000 spectrophotometer (Thermo Fisher Scientific). The cleared whole-cell lysate was further mixed with an equal volume of 2× SDS sample buffer (0.1 M Tris-HCl [pH 6.8], 4% SDS, 3.1% dithiothreitol, 20% glycerol, 0.01% Bromophenol Blue) and boiled for 15 min before loading onto a SuperSep Ace 5–20% polyacrylamide gel (Fujifilm Wako Pure Chemical Corp., Osaka, Japan); 35 μg of each sample was loaded onto the gel (Figure S3). After SDS-PAGE, proteins were transferred to Immobilon-P transfer membranes (Millipore, Billerica, MA, USA) using a PoweredBLOT 2 M apparatus (ATTO, Tokyo, Japan) and probed with a 1:1000 diluted anti-FimA primary antibody, followed by a reaction with 1:10,000 diluted peroxidase-conjugated monoclonal anti-rabbit immunoglobulins (Sigma-Aldrich Japan, Tokyo, Japan). Signals were detected using Western BLoT Chemiluminescence HRP Substrate (Takara Bio, Shiga, Japan), according to the manufacturer’s instructions. Luminescence signal images were obtained using LAS-4000 mini (FUJIFILM, Tokyo, Japan).

## 3. Results

### 3.1. Evaluation of the Mutations Introduced by Error-Prone PCR

Random mutagenesis within the *fimS* periplasmic-domain-coding region was performed by error-prone PCR, and then the periplasmic-domain-coding region of wild-type *fimS* was exchanged with randomly mutated PCR amplicons using iPCR. The DpnI-treated iPCR product was then introduced into *E. coli* chemically competent cells. A total of 514 colonies grew on the selection plates. Of these, eight colonies were randomly selected and analyzed for mutation rates. Direct DNA sequencing revealed that seven of the eight colonies had two to four (average, 2.6) nucleotide mutations within the periplasmic coding sequence, which caused 1–3 (average, 1.75) amino acid substitutions per clone. We therefore maintained this mutation condition for the subsequent construction of the *Pg* random mutant library.

### 3.2. Comparison of the Transformation Efficiency of Pg between Conjugation and Electroporation

Five days after starting the culture of conjugated AGFS1 cells on the selection plates, we found that more than 500 black-pigmented *Pg* clones had grown. However, when gene complementation of AGFS1 cells with pTCBex derivative plasmids was carried out by electroporation, only 108 transformants showed the best results in a single experiment. After repeating the same experiment several times, a total of 132 transformants were obtained using multiple trials of electroporation. The results of multiple trials in this study, together with those of a previous study using the same conjugal transfer system [13], indicated that the transformation of *Pg* with pTCBex-based plasmids is more efficient by conjugation than by electroporation.

### 3.3. Amplification of the Periplasmic-Domain-Coding Sequences from the fimS-Complemented Pg Colonies

**Figure 3:**
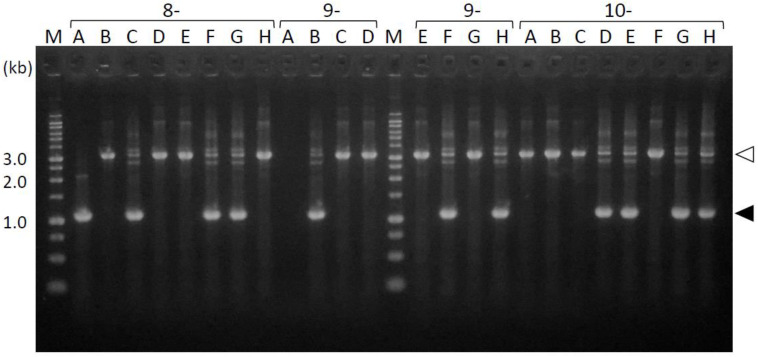
PCR amplicons of the *fimS* periplasmic-domain-coding sequences from the complemented AGFS1 colonies. The amplicons from clones 8A–10H are shown as examples. The 1.1 kb fragments (closed arrowheads) were amplified from the expression plasmids. The 3.3 kb fragments (open arrowheads) were derived from the endogenous *fimS* locus into which a 2.2 kb erythromycin cassette was inserted. M, ladder marker.

To determine the mutation sites in the transformants, we first amplified the periplasmic-domain-coding sequences directly from the *fimS*-complemented *Pg* colonies growing on agar plates. As shown in Figure 3, the 1.1 kb fragments were amplified to determine the exact size of the periplasmic-domain-coding sequences of the complemented *fimS* from the expression plasmids. In some clones, 3.3 kb fragments were amplified rather than 1.1 kb fragments; these were determined to be derived from the endogenous wild-type *fimS* locus, into which a 2.2 kb erythromycin cassette was inserted. These results demonstrated that if the introduced plasmid harboring *fimS* was kept intact in the *Pg* host, the shorter fragment of *fimS* is preferentially amplified from the plasmid but not from the endogenous locus. This phenomenon enabled the use of the PCR amplicons as templates in subsequent DNA-sequencing analysis conveniently because the additional isolation step of the target fragment can be omitted. Overall, 1.1 kb fragments were successfully amplified from 53 out of 98 clones in transformants generated by conjugation, whereas these fragments were amplified from 63 of the 132 clones for the transformants obtained by electroporation; accordingly, the efficiencies of target DNA amplification by PCR using these two transformation methods were 54% and 47%, respectively.

### 3.4. Introduced Mutations and Deduced Amino Acid Substitutions in the FimS Periplasmic Domain

DNA sequencing was performed to identify the introduced mutations and deduced amino acid substitutions in the FimS periplasmic domain, and the results are summarized in Table 2. In the transformants generated by conjugation, 18 clones were sequenced for their 1.1 kb PCR amplicons. Of these, clone F3 was found to have up to 45 mutations and 28 deduced amino acid substitutions, which was too high a number to enable the determination of the contribution of each residue to the function of FimS; thus, this clone was eliminated from further analyses. The remaining 17 clones had an average of 2 base mutations and 1.5 amino acid substitutions in the periplasmic domain, comprising 11 clones with a single substituted residue, 2 clones with two substitutions, 1 clone with three substitutions, and 3 clones with four substitutions. The clones were then subjected to phenotypic analyses. For the transformants generated by electroporation, the PCR amplicons of 57 of the 63 clones were completely sequenced. Of these, two clones had premature stop codons (clone #12, T608A mutation for L203stop and clone #99, G280T mutation for E94stop), and 34 clones had one to five amino acid substitutions. However, 22 clones were accidentally lost during subculture or freezing, and the remaining 12 clones were subjected to further phenotypic analyses. Among these clones, a single substitution was found in five clones, two substitutions were found in six clones, and five substitutions were found in one clone.

### 3.5. Expression of FimA at Transcriptional and Translational Levels in the fimS-Complemented AGFS1 Strain

**Figure 4:**
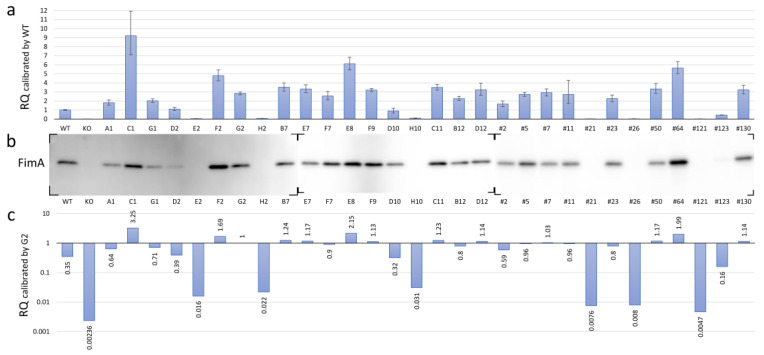
FimA phenotypes of the mutant *fimS*-complemented AGFS1 strains. (**a**) RQ-PCR results calibrated by the wild-type (WT) strain. Error bars indicate maximum and minimum values in the triplicate experiments. (**b**) Western blot using anti-FimA peptide antibodies. Total protein samples were separated by SDS-PAGE and transferred onto three independent membranes (also see Supplementary materials Figure S3). (**c**) RQ-PCR results calibrated by G2, a clone complemented with wild-type *fimS* carrying no amino acid substitutions (also see Table 3).

*Pg* modulates the expression of all components of the FimA fimbriae by receiving unknown environmental signals through the TCS protein FimS. To investigate the phenotypic changes in the FimA fimbriae caused by amino acid substitutions within the periplasmic domain of FimS, we measured and compared the transcriptional levels of *fimA* in the *fimS*-complemented AGFS1 strains using RQ-PCR analysis. When the expression level was calibrated with that of the wild-type strain, at least four strains (C1, F2, E8, and #64) showed increased levels of *fimA* expression, whereas seven strains (E2, H2, H10, #21, #26, #121, and #123) showed significantly decreased levels of *fimA* expression (Figure 4a and Table 3). Subsequent Western blot analysis supported the initial RQ-PCR results at the translational level; the signal of the FimA protein was not detected or was only detected at trace levels in strains E2, H2, H10, #21, #26, #121, and #123 (Figure 4b). However, paradoxically, many strains showed higher transcriptional levels of *fimA* than the wild type with inconsistent production levels of the FimA protein (Figure 4a,b). Strain G2 was an iPCR amplicon-transformed strain in which no amino acid substitution was observed in the complemented *fimS* on pTCBex. Thus, this strain was treated as another wild-type *fimS* strain for comparison. Interestingly, the relative transcriptional level of *fimA* in strain G2 was approximately 2.8 times greater than that of the wild-type strain (Figure 4a), which is likely attributed to the difference between the copy number of *fimS* from chromosomal DNA and the multicopy pTCBex plasmid. When the RQ-PCR data were recalibrated with strain G2, the expression levels during transcription and translation were consistent (Figure 4b,c). Therefore, we assumed that the RQ value of approximately 3 in the wild-type calibration corresponded to that of 1 in the G2 calibration and concluded that the hyper-*fimA* strains with values 1.5 times or greater than that of strain G2 were C1, F2, E8, and #64, whereas the hypo-*fimA* strains with a value half or less than that of the wild-type strain were E2, H2, H10, #21, #26, #121, and #123.

### 3.6. Functional Mapping of the FimA Phenotypes Caused by Amino Acid Substitutions in the FimS Periplasmic Domain

**Figure 5:**
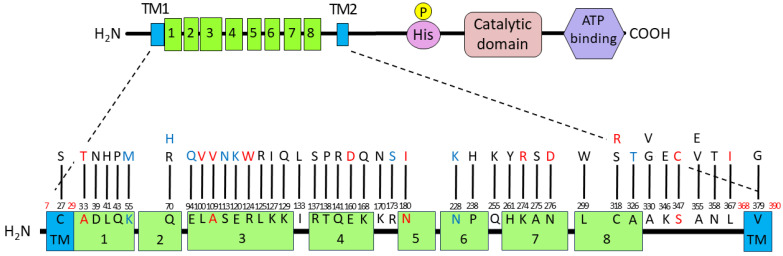
Summary of the amino acid substitutions in the FimS periplasmic domain and their resulting FimA phenotypes. The wild-type amino acid residues are numbered from the N-terminus and connected to observed replacements with bars. The substitute residues found in hyper-FimA clones are colored in blue letters, and those in hypo-FimA clones in red letters. Six amino acid residues, A33, K55, A109, N180, N228, and S347, identified from the mutants carrying only single amino acid substitutions, are also indicated in colored letters.

All amino acid substitutions and the FimA phenotypes analyzed in this study were summarized by functional mapping, which involves plotting each substituted amino acid position with phenotypic information onto the FimS periplasmic domain structure (Figure 5). Six amino acid residues, A33, K55, A109, N180, N228, and S347, were identified as critical for signal sensing, specifically the ligand binding of FimS, given that these data were obtained from the mutants carrying only single amino acid substitutions (Table 2). The substitutions A33T, A109V, N180I, and S347C resulted in FimA-negative phenotypes, indicating that these residues may be essential for direct interaction with unknown environmental signals. Although the substitutions K55M and N228K resulted in hyper-FimA phenotypes, these residues could also be important for signal sensing as they may strengthen the sensor–ligand interaction or the homodimerization of FimS monomers.

## 4. Discussion

The goal of this study was to establish a strategy for the efficient screening of the critical amino acid residues of the FimS sensor domain for sensing as-yet-unknown signals. By applying systematic protocols to construct a random mutant library, we obtained novel insights into the structure of the sensory protein dedicated to *Pg* fimbriation, confirming the usability of this approach.

Available strategies for generating mutant clones by site-directed mutagenesis are not effective and were therefore unsuitable for our goal of performing a comprehensive analysis covering the 338 amino acid residues comprising the periplasmic domain of FimS. The strategy described herein is more efficient and thereby successfully accelerated the analysis. For example, fluctuations in the expression level of FimA, either increased or decreased compared to that of the wild-type strain, caused by a single amino acid substitution strongly suggest that this residue is critical for sensing environmental signals. However, identification of the residues in the mutant strains that do not affect the expression level of FimA compared to that of the wild-type *fimS* strain is also informative because such residues could be less critical for the protein’s ligand-sensing function. Moreover, we showed the existence of such FimA-positive clones despite harboring multiple amino acid substitutions, indicating that these residues are not necessarily critical for the sensing function. This type of negative information could accelerate the comprehensive analysis of essential amino acid residues. One of the most difficult tasks in site-directed point mutation strategies is designating the appropriate residues for the replacement of the target amino acid. In fact, we showed that exchanging a target amino acid with different residues resulted in different FimA phenotypes (see Figure 5, Q70N and Q70H, C318S and C318R, as examples), which also indicates the superiority of our strategy of complementing a gene knockout strain with random mutant libraries.

In this study, we demonstrated the existence of at least six amino acid residues within the periplasmic domain of FimS that could alter the FimA phenotype if they are replaced with other residues. Interestingly, five of the six clones had single missense mutations within one of the multiple TPR motifs and exhibited either FimA-deficient or hyper-FimA phenotypes. One of the general functions of the TPR motif is to mediate protein–protein interactions [20]. Therefore, it is possible that FimS binds a polypeptide molecule as its specific substrate via the TPR motifs clustered within the periplasmic domain, and the five amino acid residues could be critical in each motif for the sensory function of FimS. To interact with FimS, which presumably penetrates the inner membrane of *Pg*, the environmental molecule must be sufficiently small to pass through the outer membrane and enter the periplasmic space.

The critical amino acid residues identified in the present study may be key residues in the hotspot of the sensor domain of FimS and may be directly associated with its unknown environmental molecules. Such structural information is useful for designing novel drugs using computational strategies [21]. These *in silico* strategies serve as alternatives to conventional “wet” experiments, such as biochemical in vitro binding assays, in which the preparation of recombinant proteins is sometimes challenging for membrane-localized sensors, including bacterial two-component sensor histidine kinases. Comprehensive isolation of critical amino acid residues in conjunction with quantitative data of FimA expression levels for each residue would be followed by functional mapping to the three-dimensional FimS sensor domain model predicted using the SWISS-MODEL service [22]. The application of a series of *in silico* molecular docking analyses can enable the screening and identification of potential lead compounds [23], which are also candidates for natural ligands of FimS. Furthermore, the reflection of the phenotypic information of the critical amino acid residues in ligand–receptor complex models could be useful for the structure-based *in silico* design of antagonists.

In conclusion, we established a series of protocols ranging from random mutagenesis of a gene of interest to the construction of a *Pg* random mutant clone library. To the best of our knowledge, this is the first report describing the application of random mutagenesis in this organism. We believe that the strategy used in this study should be generally applicable and contribute to advancing the molecular genetics research of *P. gingivalis*, especially in elucidating the structure–function relationships of other proteins of interest in this organism. As our overall goal was to evaluate the contributions of all 338 amino acid residues comprising the FimS periplasmic sensor domain to the expression of FimA, this could highlight how efficiently individual mutational data for each amino acid residue were collected. Isolating biased mutation sites is a predicted problem, and we have already been preparing to address this by modifying the composition of the error-prone PCR mixture with manganese [24]. Overall, we expect that applying these approaches will lead to the design of antagonists of FimS for novel periodontal therapy, aiming to reverse the dysbiotic changes in periodontal biofilms caused by *Pg* colonization.

## Figures and Tables

**Table 1:** Strains, plasmids, and primers used in this study.

Strain, Plasmid, or Primer	Characteristics ^1^ or Sequences (5′-3′; Amplicon Size)	Source or Reference
*Porphyromonas gingivalis* ATCC 33277 AGFS1 *Escherichia coli* DH5α BL21-Gold S17-1 Plasmids pTCBex pTCBex33277*fimS* pTCBexdm Primers ^2^ error-prone/colony PCR FimSperi5/3 direct sequencing FIMSSFW3.5 FIMSSFW4rv RQ-PCR *Pg*16S rRNA-F/R 33277 *fimA*-F/R	parent type strain (WT) 33277 with an *ermF-AM* cassete insertion in *fimS*, Em^r^ a host for general/PCR cloning a host for plasmid construction/cloning a host for conjugal transfer of pTCBex to *P. gingivalis*, Tp ^r^ a *P. gingivalis* expression vector, Ap^r^, Tc ^r^ a pTCBex carrying 33277 *fimS* ORF Mob region-deleted pTCBex ACGCGACTAACTATCCTGACATTTCTTGGA/ ATGGTTGCCACCAACAGTGTGGATATTATG; 1097 bp ATAGCCTACAACAACATGGCTAAC CATGAGACTGTTGCACATACTTACA GTCAATGGGCGAGAGCCTGAA/ AGTGTCAGTCGCAGTATGGCAA; 383 bp CTTGTAACAAAGACAACGAGGCAG/ GCAGGTGCAACGTAATTACGGCTC; 700 bp	ATCC Nishikawa and Duncan [6] Invitrogen Agilent Simon et al. [12] Nishikawa and Duncan [6], Kawamura et al. [13] Nishikawa and Duncan [6] Nishikawa [14] This study This study This study This study Nishikawa and Duncan [6] Nishikawa and Duncan [6]

^1^ Em^r^, erythromycin resistance; Tc ^r^, tetracycline resistance; Ap^r^, ampicillin resistance; Tp^r^, trimethoprim resistance. ^2^ F, forward; R, reverse.

**Table 2:** List of the *fimS*-complemented AGFS1 clones with amino acid substitutions deduced by the direct sequencing of PCR amplicons from FimS periplasmic-domain-coding region.

Strain/Clone ^1^	Mutation(s) in *fimS* Gene	Deduced Amino Acid Substitution(s) in FimS Periplasmic Domain	Accession No.
A1	G115A, A209G, T896G, T1136G	D39N, Q70R, L299W, V379G in TM2	LC800510
C1	C189A, A210C, G280C, T330G, G338A, G358A	Q70H, E94Q, S113N, E120K	LC800511
G1	A502C	K168Q	LC800512
D2	A480T, T952C, A1073C	E160D, C318R, N358T	LC800513
E2	A370C, C429T, A480T, A821G, A826G, T952C	E160D, K274R, N276D, C318R	LC800514
F2	A164T	K55M	LC800515
H2	A1039T	S347C	LC800516
B7	A1073C	N358T	LC800517
E7	T122A	L41H	LC800518
F7	C1064T	A355V	LC800519
E8	T684A	N228K	LC800520
F9	C409A	R137S	LC800521
D10	C713A, G823T	P238H, A275S	LC800522
H10	T298G, A370T	L100V, R124W	LC800523
C11	C763A	Q255K	LC800524
B12	C989T	A330V	LC800525
D12	T896G	L299W	LC800526
#2	G510T, A1036G	K170N, K346E	LC800527
#5	A385C, C1064A	K129Q, A355E	LC800528
#7	C781T, C989G	H261Y, A330G	LC800529
#11	A128C, T333C, A380T, A397C, A412C, A422G	Q43P, K127I, I133L, T138P, Q141R	LC800530
#21	A539T	N180I	LC800531
#23	C713A	P238H	LC800532
#26	C326T, T1099A	A109V, L367I	LC800533
#50	T79A	C27S in TM1	LC800534
#64	A519T, G976A	R173S, A326T	LC800535
#121	C326T	A109V	LC800536
#123	G97A	A33T	LC800537
#130	T374G, T952A	L125R, C318S	LC800538

^1^ Clones A1-D12 were plasmid-complemented by conjugation, clones #2–130 by electroporation.

**Table 3:** Relative quantification of *fimA* transcription in the *Pg* clones harboring amino acid substitutions in FimS periplasmic domain. 16S rRNA as endogenous control.

Sample ^1^	AvgΔCt ^2^	ΔCt SD	Calibrated by WT				Calibrated by G2			
			ΔΔCt	RQ	RQ Min	RQ Max	ΔΔCt	RQ	RQ Min	RQ Max
WT KO G2 A1 C1 G1 D2 E2 F2 H2 B7 E7 F7 E8 F9 D10 H10 C11 B12 D12 #2 #5 #7 #11 #21 #23 #26 #50 #64 #121 #123 #130	4.604 21.827 13.101 13.757 11.402 13.594 14.467 19.050 12.343 18.617 12.789 12.870 13.255 11.997 12.923 14.768 18.122 12.799 13.430 12.914 13.875 13.162 13.060 13.159 20.137 13.431 20.075 12.870 12.109 20.837 15.784 12.912	0.030 0.047 0.022 0.049 0.081 0.032 0.050 0.053 0.039 0.056 0.041 0.041 0.056 0.036 0.017 0.089 0.089 0.029 0.032 0.064 0.059 0.026 0.042 0.141 0.044 0.052 0.162 0.051 0.037 0.099 0.017 0.046	0 7.222 −1.504 −0.848 −3.203 −1.011 −0.138 4.446 −2.261 4.012 −1.816 −1.735 −1.350 −2.608 −1.681 0.163 3.518 −1.806 −1.174 −1.691 −0.729 −1.442 −1.544 −1.446 5.533 −1.173 5.470 −1.734 −2.496 6.232 1.179 −1.692	1 0.007 2.836 1.8 9.208 2.015 1.100 0.0459 4.795 0.062 3.521 3.328 2.549 6.095 3.207 0.893 0.087 3.496 2.257 3.229 1.658 2.718 2.916 2.724 0.022 2.256 0.023 3.327 5.641 0.013 0.442 3.232	0.941 0.006 2.691 1.538 7.114 1.821 0.938 0.0387 4.236 0.0519 3.092 2.921 2.131 5.434 3.042 0.673 0.066 3.192 2.035 2.630 1.372 2.503 2.548 1.735 0.019 1.912 0.013 2.825 5.009 0.010 0.418 2.788	1.062 0.007 2.989 2.107 11.918 2.229 1.290 0.054 5.427 0.074 4.010 3.792 3.048 6.836 3.381 1.185 0.116 3.829 2.503 3.963 2.003 2.950 3.338 4.278 0.025 2.660 0.038 3.918 6.353 0.018 0.467 3.746	1.504 8.726 0 0.656 −1.699 0.493 1.366 5.950 −0.757 5.516 −0.312 −0.231 0.154 −1.104 −0.177 1.667 5.022 −0.302 0.330 −0.187 0.775 0.062 −0.040 0.058 7.037 0.331 6.974 −0.230 −0.992 7.736 2.683 −0.188	0.353 0.002 1 0.635 3.247 0.710 0.388 0.016 1.690 0.0219 1.241 1.173 0.899 2.149 1.131 0.315 0.031 1.233 0.796 1.138 0.584 0.958 1.028 0.960 0.008 0.795 0.008 1.173 1.989 0.005 0.156 1.139	0.332 0.002 0.949 0.542 2.508 0.642 0.331 0.014 1.493 0.0183 1.090 1.030 0.751 1.916 1.073 0.237 0.023 1.125 0.718 0.927 0.484 0.883 0.898 0.612 0.007 0.674 0.005 0.996 1.766 0.003 0.147 0.983	0.375 0.003 1.054 0.743 4.202 0.786 0.455 0.019 1.914 0.026 1.414 1.337 1.075 2.410 1.192 0.418 0.041 1.350 0.882 1.397 0.706 1.040 1.177 1.508 0.009 0.938 0.013 1.381 2.240 0.006 0.165 1.321

^1^ WT, parent strain ATCC 33277; KO, *fimS*-knockout strain, AGFS1; G2, a clone complemented with wild-type *fimS* carrying no amino acid substitutions. ^2^ Average values in the triplicate experiments.

## Data Availability

The authors confirm that the data supporting the findings of this study are available within the article and its Supplementary Materials.

## Supplementary Materials

The following supporting information can be downloaded at https://www.mdpi.com/article/10.3390/pathogens13040309/s1: Figure S1: Error-prone PCR: Amplification of megaprimers used in the inverse PCR; Figure S2: Construction of expression vectors used in this study; Figure S3: The polyacrylamide gels used in the Western blot analysis.
