# Supplementary material for: Identification of Critical Amino Acid Residues of a Two-Component Sensor Protein for Signal Sensing in Porphyromonas gingivalis Fimbriation via Random Mutant Library Construction
Source: Pathogens. 2024 Apr 10;13(4):309. doi: 10.3390/pathogens13040309 (PMC11053733; doi:10.3390/pathogens13040309)
Supplement: Supplementary file 1 [file pathogens-13-00309-s001.zip › Figure S2.pdf]

Figure S2. Construction of expression vectors used in this study.

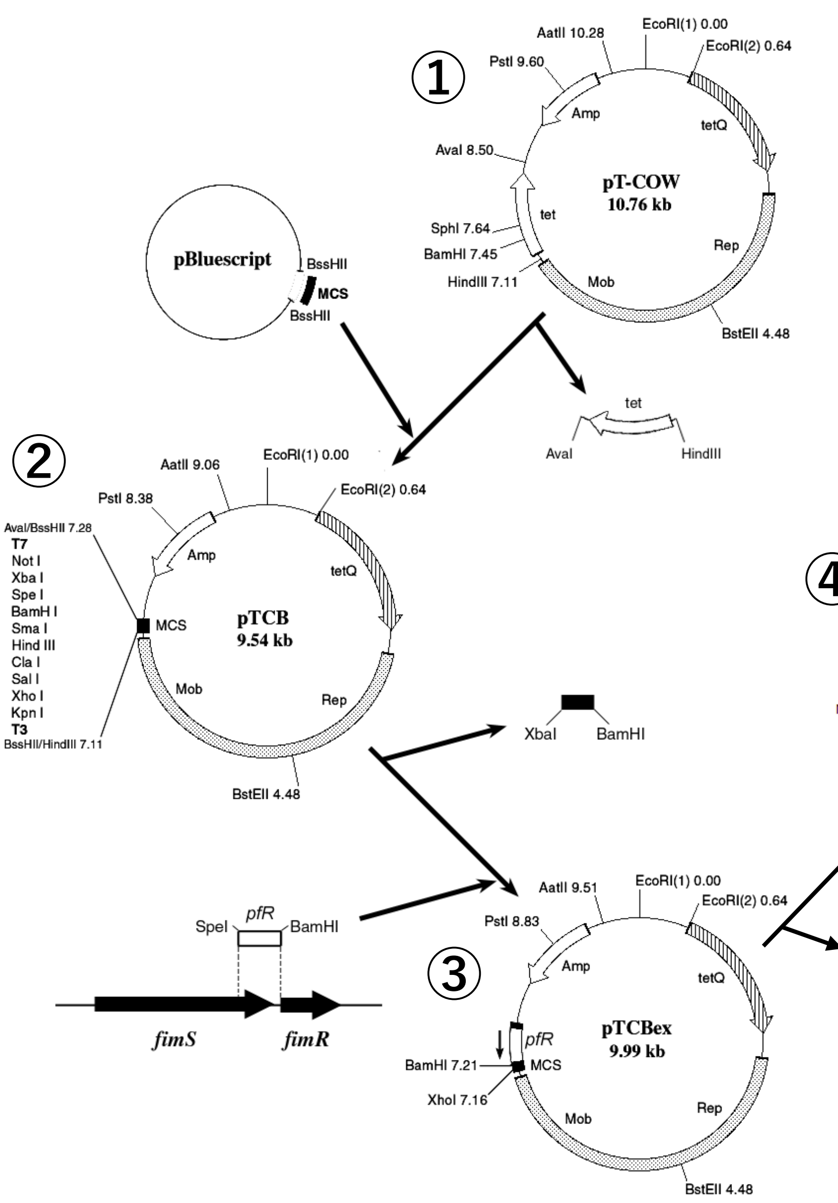

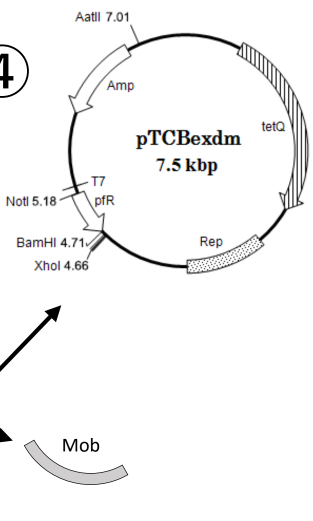

① **pT-COW**: A derivative of a plasmid from *Bacteroides*. Mobilizable into *P. gingivalis* from *E. coli* S17-1 by conjugation (Gardner et al, 1996. Appl Environ microbiol, 62: 196-202).

② **pTCB**: The tet region in pT-COW has been replaced with pBluescript MCS fragment.

③ **pTCBex**: pfR, a promoter region from Pg, has been inserted upstream of the MCS in pTCB (Nishikawa & Duncan, 2010. J Bacteriol, 192: 1975-1987).

④ **pTCBexdm**: The Mob region has been deleted from pTCBex. Introduced into Pg only by electroporation.
