# Supplementary material for: Identification of Critical Amino Acid Residues of a Two-Component Sensor Protein for Signal Sensing in Porphyromonas gingivalis Fimbriation via Random Mutant Library Construction
Source: Pathogens. 2024 Apr 10;13(4):309. doi: 10.3390/pathogens13040309 (PMC11053733; doi:10.3390/pathogens13040309)
Supplement: Supplementary file 1 [file pathogens-13-00309-s001.zip › Figure S3.pdf]

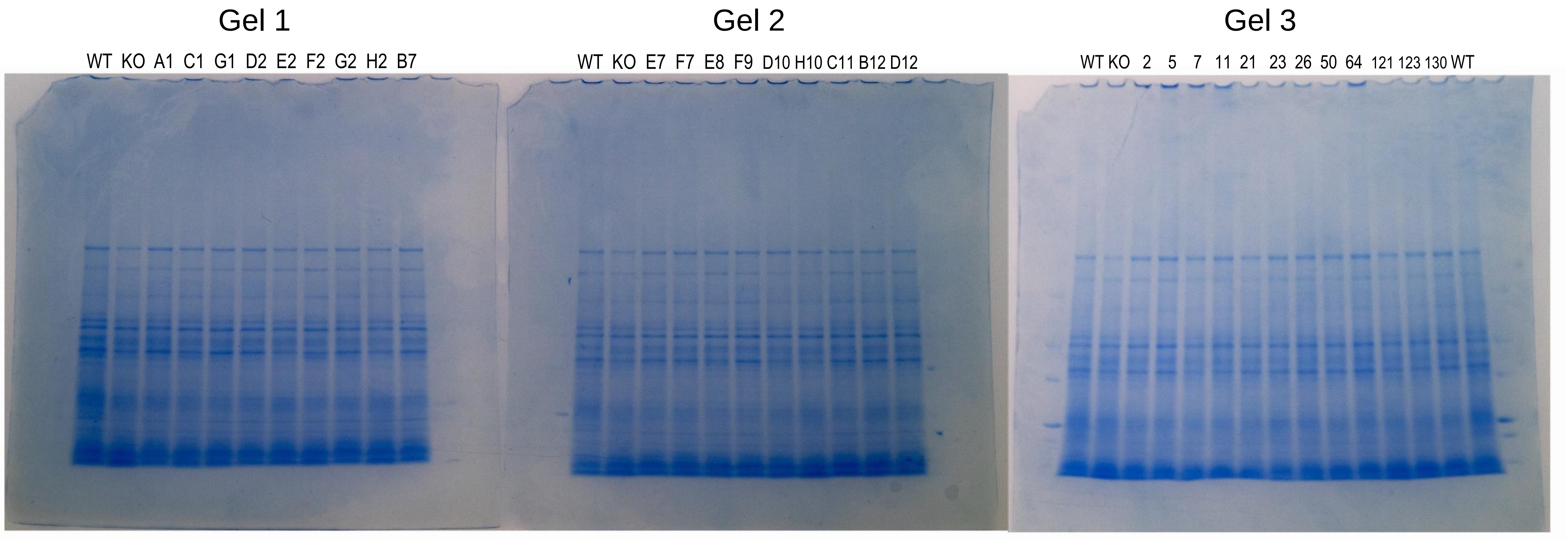

Figure S3: The polyacrylamide gels used in the western blot analysis.

Thirty five micrograms of each *P. gingivalis* whole-cell lysate was loaded onto the SDS polyacrylamide gel. After SDS-PAGE and electro-blotting, these used gels were stained with Coomassie Brilliant Blue to confirm the loading amount of proteins.

Samples from mutant clones A1-B7 were loaded onto Gel 1, E7-D12 onto Gel 2, and #2-130 onto Gel 3. WT, wild-type strain; KO, *fimS* knockout strain AGFS1. The WT and KO signals on the western blots from the gels 2 and 3 were cut off to integrate these three blots in Fig. 4.
