# Supplementary figures and images for: Identification of Critical Amino Acid Residues of a Two-Component Sensor Protein for Signal Sensing in Porphyromonas gingivalis Fimbriation via Random Mutant Library Construction
Source: Pathogens. 2024 Apr 10;13(4):309. doi: 10.3390/pathogens13040309 (PMC11053733; doi:10.3390/pathogens13040309)

Figure S1: Error-prone PCR: Amplification of megaprimers used in the inverse PCR.

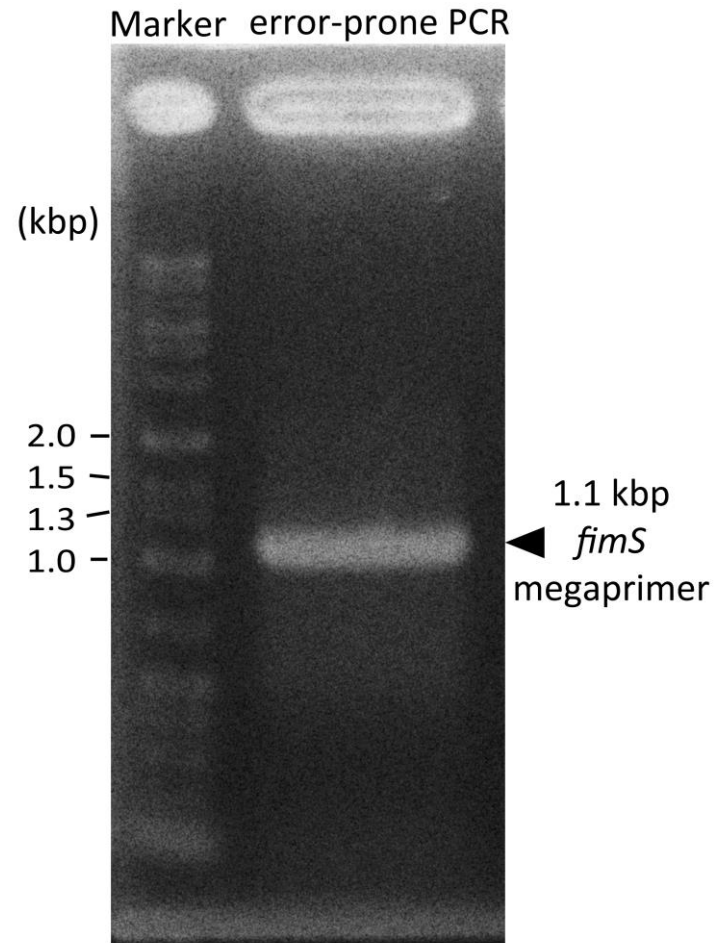

Supplement: Supplementary file 1 [file pathogens-13-00309-s001.zip › Figure S1.pdf]
